# The British Pharmaceutical Conference

**Published:** 1911-07-29

**Authors:** 


### THE BRITISH PHARMACEUTICAL CONFERENCE.

Among the papers read and discussed at the forty-eighth annual meeting of the British Pharmaceutical Conference, which was held at Portsmouth on July 25 and 26, were several of interest to medical practitioners, while others afforded useful material for hospital dispensers. The address of the President, Mr. W. F. Wells, dealt mainly with the laws relating to the practice of pharmacy, and he showed that by comparison with the German and French laws relating to dispensing and the sale of poisons, our own legislation is singularly defective. He also referred to the fact that Irish Boards of Guardians buy their drugs in the cheapest market, with the result that the drugs are of inferior quality. Some useful information was contained in a paper by Mr. F. W. F. Arnaud, F.I.C., on "The Composition of Diabetic Foods." The term "diabetic foods" embraces a wide class of products, but the author dealt only with gluten bread and flours. His investigations reveal an extremely unsatisfactory state of affairs, to which the attention of practitioners of medicine should be directed. Twelve different samples of the foods, the product of seven manufacturers, were examined, and the products of one manufacturer alone may be said to be satisfactory; in fact, a sample of one expensive diabetic food was found to consist of ordinary flour which had merely been heated. Most people will agree with the author that some steps should be taken for the repression of the business carried on in the sale of ordinary bread and flour as specially recommended diabetic foodstuffs; failing that, the composition of these foodstuffs should certainly be declared to the purchaser. Another communication of some interest to practitioners was that in which Mr. H. Deane, B.Sc., F.I.C., demonstrated the variability of extract of Indian hemp as supplied by manufacturers. The strength of the preparations examined differed to a wide extent, and there can be little doubt that this is bad for the medical man as well as for the patient. The author suggested a method by which more satisfactory results may be obtained. Another medicinal product for which it is desirable that a standard should be set up is thyroid powder, and Mr. R. R. Bennett, B.Sc., F.I.C., suggested in an interesting paper what this standard should be. He showed by the results of the examination of a number of samples of *Thyroideum Siccum* that the widespread belief among clinicians that the commercial thyroid preparations vary considerably in their degree of activity is well founded, for the combined iodine present in different preparations differs to a rather wide extent. The agreement among the majority of pharmacologists that the activity of thyroid is dependent upon the combined iodine present suggests that an iodine standard should be fixed, and the author came to the conclusion, as the result of his experiments, that an iodine standard of 0.15 per cent. might be adopted without in any way unduly harassing the manufacturer.

Hospital dispensers will be interested in the suggestion made in a paper by Mr. D. B. Dott, F.R.S.E., that instead of keeping a stock of spirit of nitrous ether, it would be better to make the preparation, when required for use, by mixing ½ fluid drachm of solution of sodium nitrite with 7½ fluid drachms of alcohol (acidified with lactic acid) to make 1 fluid ounce of spirit of nitrous ether. The deterioration of this product has always been a perplexing problem for dispensers, and the author's suggestion is worthy of consideration, especially in view of the fact that the Pharmacopœia is in course of revision. Another suggestive paper was read by Mr. F. H. Alcock, F.I.C., who proposed an improved method of preparing liniment of ammonia. When made according to the official process this product sometimes becomes solid on keeping. To avoid this the author suggested that it should be made according to the following formula: Almond oil 3 fluid ounces, olive oil 8 fluid ounces, strong solution of ammonia (0.880), 1 fluid ounce. The product is not so elegant as the fresh official preparation, but it is very effectual. The author also suggested that in a future Pharmacopœia the formula should contain no almond oil, but olive oil only, as almond oil is more expensive. A new method of making spirit of sal volatile was described by Mr. G. W. Pollard, B.Sc. The process is as follows: Oil of nutmeg 4½ fluid drachms, oil of lemon 6½ fluid drachms, water 2 pints—distil 1 pint and mix it with 6 pints of alcohol; dissolve, by the aid of a little gentle heat, 4 ounces of ammonium carbonate in 8 ounces of strong solution of ammonia and 9 ounces of water. Add this solution to the alcoholic solution of the oils. The author finds that this process gives a spirit equal in all respects to that prepared by the official process, and dispensers will find that small apparatus such as is used for analysis is quite sufficient for a gallon of the preparation.

The above subjects were among those dealt with in the Science section, but in the Practical section matters of a different character were discussed, such as the position of pharmacists in relation to the proposed scheme of national insurance, secret remedies, and pharmaceutical education. The discussion on "Secret and Proprietary Remedies" was opened by Mr. E. J. Harrison, B.Sc., F.I.C. Referring to the suggestions that have been made in regard to new legislation on this subject, he pointed out that the one which had found most favour with the medical profession was that the law should make it compulsory for all medicines advertised or sold for the cure of disease, when not supplied to a medical prescription, to bear on the label a full statement of their exact composition, and that the label should have the force of a warranty, with penalties for misstatements. Proprietary medicines would then be in the same position as other medicines and foods now are under the Sale of Food and Drugs Acts, and the purchaser would be protected from misdescription and fraud in the same way as he is with other articles, and as he certainly ought to be in a civilised community. In the opinion of Mr. Harrison, however, this proposal goes a little too far, and he suggested that it would be enough to require the declaration on the label of the names of all the principal ingredients and the exact quantities of any coming within the poisons schedule or possessing great activity. Legislation on such lines would make it impossible for the fraudulent quack medicines to flourish; these nostrums depend for their existence not on any special skill in compounding or on any knowledge of the best combinations which it is desirable to keep secret from competitors, but in keeping secret from the public the fact that their composition is such that they cannot possibly cure the diseases named in the advertisements. The medical profession can certainly depend upon the support of the British Pharmaceutical Conference in their attempt to restrict the sale of quack medicines.

